# Rutin mitigates perfluorooctanoic acid-induced liver injury via modulation of oxidative stress, apoptosis, and inflammation

**DOI:** 10.22038/IJBMS.2023.69747.15187

**Published:** 2023

**Authors:** Maloos Naderi, Mohammad Seyedabadi, Fereshteh Talebpour Amiri, Sholeh Akbari, Fatemeh Shaki

**Affiliations:** 1 Department of Toxicology and Pharmacology, Faculty of Pharmacy, Mazandaran University of Medical Sciences, Sari, Iran; 2 Student Research Committee, Mazandaran University of Medical Sciences, Sari, Iran; 3 Pharmaceutical Sciences Research Center, Faculty of Pharmacy, Mazandaran University of Medical Sciences, Sari, Iran; 4 Department of Anatomy, Molecular, and Cell Biology Research Center, Faculty of Medicine, Mazandaran University of Medical Sciences, Sari, Iran; 5 Pharmaceutical Sciences Research Center, Hemoglobinopathy Institute, Mazandaran University of Medical Sciences, Sari, Iran

**Keywords:** Apoptosis, Hepatotoxicity, Inflammation, Oxidative stress, Perfluorooctanoic acid, Rutin

## Abstract

**Objective(s):**

Perfluorooctanoic acid (PFOA) is a persistent organic pollutant (POP), broadly present in the environment. Due to long biological half-life, it is accumulated in the body, especially the liver, causing hepatocellular damage. This study was designed to assess the effects of rutin on PFOA-induced liver damage in rats.

**Materials and Methods:**

Male Wistar rats were exposed to PFOA (10 mg/kg/day) alone, or in combination with different doses of rutin (25, 50, and 100 mg/kg/day) by oral gavage for 4 weeks.

**Results:**

PFOA altered the levels of liver enzymes, induced a notable change in the tissue structure of the liver, caused some levels of mitochondrial dysfunction, and increased the expression of pro-apoptotic and pro-inflammatory genes. Co-treatment with rutin mitigated the PFOA-induced elevation of liver enzymes, histopathological defects, oxidative damage, and mitochondrial dysfunction. In addition, rutin declined the stimulatory effects of PFOA on the Bax: Bcl2 ratio and reduced the PFOA-induced gene expression of TNF-α, IL-6, NF-ƙB, and JNK.

**Conclusion:**

These findings suggest rutin as a protective agent for PFOA-induced liver injury, albeit the protection was partial. Possible mechanisms are inhibition of oxidative stress, mitochondrial dysfunction, and inflammatory response.

## Introduction

Perfluorooctanoic acid (PFOA), as a stable synthetic organic compound, is extensively used for the production of a large variety of consumer products and industrial processes (1). PFOA, due to carbon-fluorine bonds, is extremely resistant to environmental degradation and is accumulated in the food chain, causing the greatest ecological concern (2). Previous research confirmed that exposure to PFOA results in various toxicities (3). In this regard, the liver is the major organ for the accumulation as well as toxicity of PFOA, albeit the exact mechanisms are yet to be elucidated. 

Oxidative stress (4) and mitochondrial dysfunction (5) are the main mechanisms by which environmental pollutants can cause liver injury. In fact, xenobiotics induce mitochondrial dysfunction, leading to excessive production of reactive oxygen species (ROS), loss of mitochondrial membrane potential, and finally apoptosis (6). Recent studies have verified that PFOA causes cellular damage and mitochondrial defect in pancreatic β-cells (7).

Inflammation is another theory associated with hepatotoxic response (8-10). Exposure to PFOA elevates the production of pro-inflammatory cytokines such as IL-6 and TNF-α (11). Nuclear Factor Kappa B (NF-κB), the main transcriptional regulator of inflammation, is also involved in stress response and innate immunity. Its activation leads to the transcription of genes involved in the synthesis of inflammatory cytokines (12, 13). c-Jun-N-terminal kinase (JNK) is also linked to NF-κB and plays a significant role in inflammatory responses (12). Multiple stimuli associated with inflammation (for instance, TNF-a, IL-1, and oxidative stress) activate both JNK/AP-1 and NF-kB (14, 15).

Rutin (3-rhamnosyl-glucosyl quercetin), is a flavonoid glycoside abundantly found in certain vegetables and fruits (16). The antioxidant and anti-inflammatory properties of rutin are shown to protect vital organs from the deleterious effects of ROS (17). In particular, rutin binds to iron ions, and inhibits the interaction of hydrogen peroxide in Fenton reaction, thus preventing one of the key steps in the production of reactive free radicals (18). Rutin attenuated isoniazid-induced liver injury via inhibition of oxidative stress (19). The current investigation was designed to evaluate the potential protective effects of rutin on PFOA-induced hepatotoxicity and its possible molecular mechanisms by assessing oxidative stress, mitochondrial dysfunction, and inflammation in male rats.

## Materials and Methods 

***Animal treatment and experimental design***

PFOA and rutin were purchased from Sigma Aldrich (St. Louis, MO, USA). PFOA was dissolved in water and administered orally by gavage at the dose of 10 mg/kg, as reported in previous studies (20) with some changes based on our pre-test data. The dose of rutin used in this study was determined according to previous studies (17). Rutin was dissolved in 1% DMSO and administered orally by gavage for 1 hr prior to PFOA administration.

Male Wistar rats (200-250 g) were obtained from the Laboratory Animal Research Center of Mazandaran University of Medical Sciences, Sari, Iran. All experimental procedures were approved by the ethical committee of Mazandaran University of Medical Sciences, Sari, Iran (Ethical code: IR.MAZUMS.REC.1399.6354). After acclimatization, rats were randomly divided into seven groups (n=6), and treated once daily by gavage for 4 weeks as follows: 1) control (normal saline); 2) rutin (100 mg/kg); 3) PFOA (10 mg/kg); 4-6) PFOA (10 mg/kg)+different doses of rutin (25, 50, and 100 mg/kg); and 7) PFOA (10 mg/kg)+vitamin C (100 mg/kg) as a positive control.

At the end of treatment, the animals were anesthetized (xylazine 5 mg/kg and ketamine 80 mg/kg), and blood samples were collected for biochemical assays. The liver was separated and split into three parts and stored in suitable conditions for histopathological, gene expression, and mitochondrial studies (21).

***Serum biochemical evaluation***

The serum levels of alanine transaminase (ALT) and aspartate aminotransferase (AST) were estimated by employing commercial standard kits (Pars Azmun, Iran) with Alpha-Classic Auto Analyzer. 

***Histopathological examination***

Liver samples were fixed, processed, sectioned, and stained with hematoxylin and eosin (H&E) according to the protocols described in our previous study. The degree of liver injury was then determined under light microscopy. Liver damage was determined as the sum of all manifestations (22).

***Assessment of oxidative stress in liver tissue***

*Total protein*

Protein concentrations were determined in samples through the Coomassie Blue protein binding Bradford method (23).

*Liver oxidative stress*

*Reactive oxygen species (ROS)*

The study measured intracellular levels of ROS in liver homogenates using the dichlorodihydro- fluorescein diacetate (DCFH-DA) dye as an indicator. The liver homogenate was diluted with phosphate-buffered saline (PBS) to a ratio of 1:20 (v/v), followed by mixing 190 μl of the homogenate with 10 μl of 1 mM DCFH-DA. After incubating for 30 min at 37 °C, the conversion of DCFH-DA to 2′, 7′-dichlorofluorescein was measured using a JASCO FP-6200 spectrofluorometer at excitation/emission wavelengths of 485/520 nm, respectively. ROS concentration was reported as fluorescent intensity per 1 mg protein (24). 

*Lipid peroxidation (LPO)*

Malondialdehyde (MDA) formation was determined as an indicator for lipid peroxidation, utilizing thiobarbituric acid (TBA) as a marker. The absorbance of the supernatant was measured using an ELISA reader at the wavelength of 532 nm (Tecan, Rainbow Thermo, Austria) to evaluate the quantity of MDA formation. The standard is Tetramethoxypropane and results were expressed as micromolar (μM)/mg protein (25). 

*Protein carbonyl (PrC)*

The protein carbonyl level was evaluated by the spectrophotometric method at 365 nm. Briefly, samples were treated with TCA and incubated at 4 ^°^C for 15 min. The precipitates were incubated with 2, 4-dinitrophenylhydrazine (DNPH) at room temperature for 1 hr, while vortexing was performed every 5 min. After protein precipitation and dissolution in guanidine hydrochloride, the carbonyl content was measured at 365 nm (26).

*Reduced glutathione reduction (GSH)*

Estimation of reduced glutathione content in liver tissue homogenates was performed using dithiobis (2-nitrobenzoic acid) (DTNB) as an indicator, and the yellow color that developed was read at 412 nm on a spectrophotometer (UV-1601 PC, Shimadzu, Japan). The results were expressed in μM/mg protein (27).

*Superoxide dismutase (SOD) activity*

SOD activity assay was measured using Nasdox commercial kit (Navand Salamat Co, Iran). The assay is based on the inhibition of the oxidation of pyrogallol, which is normally oxidized in the presence of o-2. The presence of SOD inhibits the autooxidation of pyrogallol, allowing for indirect assessment of enzyme activity using a spectrophotometer at 405 nm (26). In essence, the activity of SOD was quantitatively determined using a commercially available kit that measures the inhibition of pyrogallol oxidation (26).

*Nitric oxide* *(NO) level*

The concentration of nitric oxide was measured using a rat-specific ELISA kit (Cib Zist Fan Co., Iran) based on the Griess reaction. In the first step, nitrite reacts with sulfanilic acid to form diazonium ions. In the second step, these ions are combined with N-(1-naphthyl) ethylene diamine, resulting in the formation of a pink-colored azo compound. This reaction is well-established in scientific literature and has been previously described (26). 

***Mitochondrial function***

*Mitochondrial viability*

Mitochondrial viability was assayed using 3-(4,5-dimethylthiazol-2-yl)-2,5-diphenyltetrazolium bromide (MTT). Briefly, MTT (0.4%) was added to 100 μl of fresh isolated mitochondrial suspensions (1 mg protein/ml) and then incubated at 37 ^°^C for 30 min. The plate was read at 570 nm after dissolution of purple formazan crystals with DMSO (26, 28).

*Mitochondrial membrane potential (MMP)*

The measurement of mitochondrial membrane potential (MMP) was determined by the uptake of cationic fluorescent dye, rhodamine-123, into the mitochondria. To conduct the MMP assay, a mitochondrial fraction solution containing 1 mg protein/ml was prepared with the addition of 10 μM rhodamine 123 in MMP assay buffer, which consisted of various components such as sucrose, D-mannitol, KCl, KH_2_PO_4_, MgCl_2_, EGTA, sodium succinate, HEPES, and rotenone. The fluorescence intensity of rhodamine released outside the mitochondria was detected using a fluorescence spectrophotometer (Shimadzu RF5000U) at the excitation and emission wavelengths of 490 nm and 535 nm, respectively. An increase in fluorescence intensity indicates a decrease in MMP.(29, 30).

*Mitochondrial swelling*

The evaluation of mitochondrial swelling was carried out by observing changes in light scattering measured at 540 nm using a spectrophotometer. Initially, freshly isolated mitochondria were suspended in a buffer designed to promote swelling, which consisted of 70 mM sucrose, 230 mM mannitol, 3 mM HEPES, 2 mM Tris-phosphate, 5 mM succinate, and 1 μM rotenone. The absorbance of the mitochondrial suspension was measured using an ELISA reader manufactured by Tecan; Rainbow Thermo, Austria. A decrease in absorbance is accompanied by an increase in mitochondrial swelling (26).

***RNA extraction and real-time polymerase chain reaction (RT-PCR)***

Total RNA was extracted from liver tissue using Hybrid-R™ total RNA isolation kit (Denazist, Iran) according to the manufacturer’s instructions. cDNA synthesis was accomplished using a Pars-Tous commercial kit (31, 32). Real-time PCR was performed with specific primers (list in supplementary file) for each gene using Corbett Rotor-Gene 6000 (Qiagen, Germany) and normalized to glyceraldehyde-3-phosphate dehydrogenase (GAPDH) levels, with the 2 ^−ΔΔCT^ method (33).

***Statistical analysis***

The mean and standard deviation (SD) were used to express the data. Statistical analysis was performed using one-way ANOVA with Tukey’s *post hoc* test for parametric variables and Kruskal-Wallis H test for non-parametric data. The Shapiro-Wilk test was employed to assess the normality assumption. GraphPad Prism version 8 was utilized for both data presentation and analysis, and statistical significance was considered at *P*-value<0.05.

## Results

***Serum biochemical evaluation***

As summarized in Table 1, the PFOA group showed a marked increase in liver enzymes (*P*<0.001). In fact, AST and ALT increased by 53 and 19 units, respectively, in rats treated with PFOA compared to the control group. Rutin supplementation alone caused no significant change in liver enzymes. However, Animals co-treated with PFOA and 50 or 100 mg/kg of rutin, or PFOA and vitamin C demonstrated significantly lower levels of these enzymes, in comparison with the PFOA group.

***Histopathological observations***

**Figure 1:**
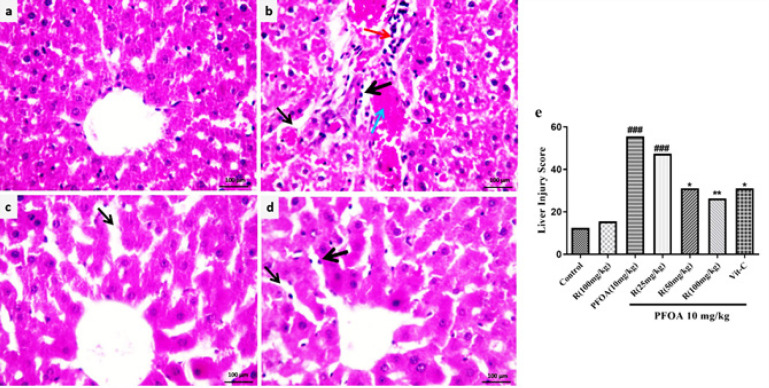
Photomicrographs show the effect of R (Rutin) on the histological architecture of the liver in PFOA (perfluorooctanoic acid) treated rats

Normal liver architecture was observed in the control group However, extensive morphological changes including Kupffer cell proliferation, infiltration of the inflammatory cell, hemorrhage, and dilation of sinusoids were observed in the PFOA-treated group. Co-treatment with rutin alleviated the pathological changes induced by PFOA. Liver injury’s mean grading scores are presented in Figure 1e. Liver injury score was elevated in PFOA-treated rats, which was lower in the rutin plus PFOA group compared to the PFOA group (Figure 1).

***Oxidative stress in liver tissue***

Exposure to PFOA showed a significant increase in ROS formation, MDA, protein carbonyl levels, and nitric oxide concentration, while GSH content and SOD activity were decreased in comparison with the control group (*P*<0.001), as shown in Table 2. Co-treatment with rutin at 50 and 100 mg/kg showed a significant decrease in ROS formation, MDA, protein carbonyl levels, *and *nitric oxide concentration compared to the PFOA group. Rutin at 50 (*P*<0.01) and 100 (*P*<0.001) mg/kg reversed the effects of PFOA on GSH content. Co-treatment with 50 (*P*<0.05) and 100 (*P*<0.01) mg/kg of rutin showed a significant increase in SOD activity by 1.1 and 1.5 U/ml, respectively, compared to the PFOA group. Also, vitamin C alters all mentioned factors except PrC levels. Treatment with rutin alone had no effect on the above parameters compared with the control group. 

***Mitochondrial function assay***

*Mitochondrial viability*

**Figure 2:**
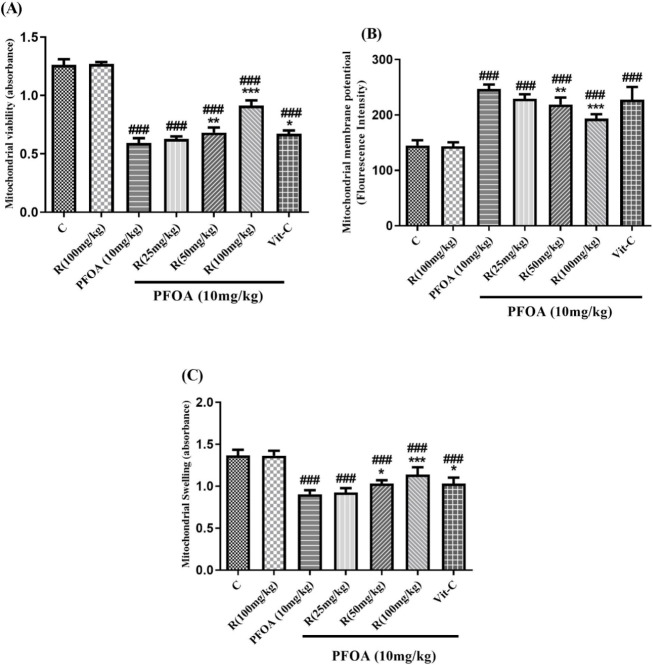
Effect of rutin on (a) mitochondrial function, (b) mitochondrial membrane potential, and (c) mitochondrial swelling in PFOA-treated rats

PFOA decreased mitochondrial viability in the liver by 66% compared to the control. Co-treatment with 50 (*P*<0.01) and 100 (*P*<0.001) mg/kg of rutin or vitamin C (*P*<0.05) significantly attenuated PFOA-induced mitochondrial dysfunction. The treatment with rutin alone had no effect on mitochondrial viability compared with the control group (Figure 2a). 

*Mitochondrial membrane potential*

An increase in rhodamine fluorescence (MMP collapse) was more than doubled in PFOA-treated animals in comparison with the control group. Besides, co-treatment with 50 (*P*<0.01) or 100 mg/kg (*P*<0.001) of rutin but not vitamin C significantly reversed the PFOA-induced MMP collapse (Figure 2b).

*Mitochondrial swelling*

As shown in Figure 2c, exposure to PFOA resulted in a significant increase in mitochondrial swelling by 46% in comparison with the control group. However*,* administration of rutin at doses of 50 (*P*<0.05) or 100 (*P*<0.01) mg/kg as well as vitamin C (*P*<0.05) significantly decreased mitochondrial swelling caused by PFOA. 

***TNF-α*** *,* ***IL-6*** *,* ***NF-*** ***ƙ*** ***B*** *,* ***JNK*** *,* ***Bax*** *,* ***and Bcl-2 genes expression***

**Figure 3:**
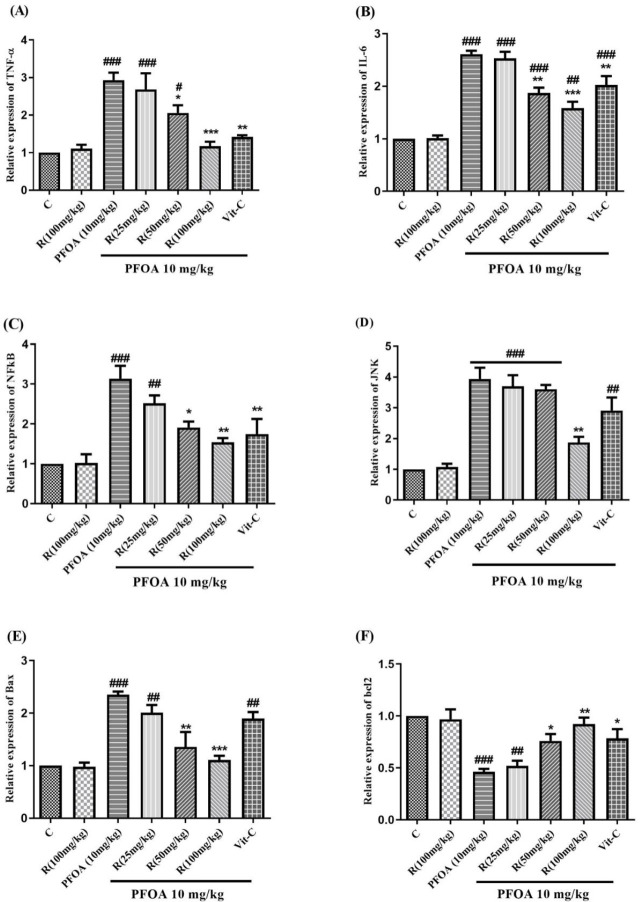
Effect of treatments on (a) TNF-α, (b) IL-6, (c) NF-ƙΒ, (d) JNK, (e) Bax, and (f) Bcl-2 gene expression in rat liver tissue

According to the RT-PCR analysis, the gene expression of TNF-α, IL-6, NF-ƙB, and JNK increased by 1.9, 1.6, 2.1, and 2.9 times, respectively, in rats treated with PFOA compared to the control group. Co-treatment with 50 or 100 mg/kg of rutin as well as vitamin C (*P*<0.05) attenuated PFOA-induced increase of TNF-α, IL-6, NF-ƙB gene expressions. However, only high-dose rutin was capable of mitigating the PFOA-induced JNK expression. Rats exposed to rutin alone did not show a significant difference in the expression of TNF-α, IL-6, NF-ƙB, and JNK, compared with the control group (Figure 3). According to the RT-PCR analysis, the gene expression of Bax was more than doubled, and the gene expression of Bcl-2 was almost halved in animals treated with PFOA (*P*<0.001), compared to the control. Co-treatment with rutin at 50 or 100 mg/kg decreased the PFOA-induced increase of the Bax: Bcl-2 ratio. However, rutin treatment did not affect the Bax: Bcl-2 ratio, per se (Figure 3).

(a) control; (b) PFOA; (c) PFOA+R 50 mg/kg; (d) PFOA+R 100 mg/kg; and (e) Liver injury scores. Thick black arrow: Kupffer cell proliferation; Red arrow: Infiltration of inflammatory cell; Blue arrow: hemorrhagic; and thin black arrow: Dilation of sinusoids. Treatment with rutin ameliorated these changes. H&E staining; Mag: ×40; Scale bar =100 μm. The highest score belongs to the PFOA group. Rutin was able to reduce liver injury scores in the PFOA+R group

^###^ Significantly different in comparison with the control group. * *P<*0.05 and ** *P<*0.01 significantly different from PFOA groups

**Table 1:** Effect of rutin on liver enzymes in PFOA-treated rats

Groups	AST(U/L)	ALT(U/L)
**Control** **R(100 mg/kg)**	145±9.2 144±4.8	60.3±3.5 61.6±1.7
**PFOA(10 mg/kg)**	198.1±9.37^###^	79.3±5.9^###^
**PFOA+R (25 mg/kg)**	193±8.5^###^	79±3.52^###^
**PFOA+R (50 mg/kg)**	181.6±3.25^*^^,###^	72±3.03^*^^,###^
**PFOA+R (100 mg/kg)**	168±8.21^***^^,###^	68.8±3.37^***^^,##^
**PFOA+Vit-C**	178.1±9.37^**^^,###^	71.3±3.83^*^^,###^

Data are expressed as Mean±SD for six rats in each group and analyzed by ANOVA followed by Tukey’s test. ^##^*P<*0.01 and ^###^*P<*0.001, in comparison with control group. *P<0.05, **P<0.01, and ***P<0.001, in comparison with PFOA (10 mg/kg) group

PFOA: perfluorooctanoic acid; R: Rutin

**Table 2:** Effect of rutin on biomarkers of oxidative stress and NO level in PFOA-treated rats

Groups	ROS formation (fluorescence intensity)	LPO (µmol/mg proteins)	GSH (µmol/mg proteins)	PrC (mmol/mg proteins)	SOD activity (U/ mg proteins)	NO (nmol/ml)
**Control** **R(100 mg/kg)**	49.9 ±5.46 48.6±8.67	11.9 ±0.78 11.4±0.71	207±11.5 203±9.88	0.39 ±0.03 0.37±0.05	11.1 ±1.14 11.7±0.38	34.6 ±3.08 33.4±1.46
**PFOA ** **(10 mg/kg)**	92.5 ±7.61^###^	18.8 ±0.78^###^	178 ±9.11^###^	0.59 ±0.03^###^	7.78 ±0.33^###^	71.4 ±4.77^###^
**PFOA+R(25 mg/kg)**	85.6 ±4.24^###^	18.2 ±0.26^##^	196 ±8	0.54 ±0.02^###^	7.99 ±0.71^###^	65.7 ±2.27^###^
**PFOA+R(50 mg/kg)**	65.6±7.80^***^^,^^##^	17±0.85^**^^,^^###^	201 ±8.54^**^	0.51±0.04^**^^,^^###^	8.96 ±0.3^*^^,^^###^	62 ±3.62^**^^,^^###^
**PFOA+R(100 mg/kg)**	57.9 ±5.57^***^	13.6 ±0.73^***^^,^^##^	205 ±12.9^***^	0.49±0.05^***^^,^^## ^	9.3 ±0.26^**^^,^^###^	56.9 ±4.83^***^^,^^###^
**PFOA+Vit-C**	80.1 ±5.45^*^^,^^###^	17 ±0.81^**^^,^^###^	199 ±9.42^*^	0.56 ±0.03^###^	8.93 ±0.57^*^^,^^###^	63.7 ±3.92^*^^,^^###^

Data are expressed as Mean±SD for six rats in each group and analyzed by ANOVA followed by Tukey’s test. ^##^*P<*0.01 and ^###^*P<*0.001, in comparison with the control group. *P<0.05, **P<0.01, and ***P<0.001, in comparison with PFOA (10 mg/kg) group

PFOA: perfluorooctanoic acid; R: Rutin; ROS: reactive oxygen species; LPO: Lipid peroxidation; GSH: Reduced glutathione reduction; PrC: Protein carbonyl; SOD: Superoxide dismutase activity; NO: Nitric oxide

Data are expressed as mean±SD. ^###^*P<*0.001, significantly different in comparison with the control group. * *P<*0.05, ** *P<*0.01, and ***P<0.001, significantly different in comparison with PFOA (10 mg/kg) group

PFOA: perfluorooctanoic acid; R: Rutin

GAPDH served as an internal standard. Data are expressed as mean±SD. ^#^*P<*0.05, ^##^*P<*0.01, and ^###^*P<*0.001, in comparison with the control group. *P<0.05, **P<0.01, and ***P<0.001 were significantly different in comparison with PFOA (10 mg/kg) group

PFOA: perfluorooctanoic acid; R: Rutin

## Discussion

PFOA is a durable chemical commonly used in numerous applications in industrial processes. Recently, there has been increasing global concern about the environmental and health risks of PFOA. It has been shown that exposure to PFOA is associated with an array of human diseases, including hepatic injury which is marked by liver enlargement and fatty liver (34). 

Liver damage was proved by a significant rise in ALT and AST levels in rats treated with PFOA, which is consistent with previous reports (1). Histopathological examination of liver sections in PFOA-treated rats also revealed various morphological changes. As shown in the results, the increase in liver enzymes (Table 1) and morphological defects (Figure 1) were significantly less in rats co-treated with PFOA and rutin or PFOA and vitamin C compared to those receiving only PFOA, although they were still higher than in the control group. These findings suggest that the administration of rutin or vitamin C alongside PFOA may confer some protective effect against PFOA-induced liver damage.

The Role of oxidative stress in PFOA-induced hepatotoxicity was exhibited by enhanced ROS production, increased lipid peroxidation, reduced GSH content, and decreased activity of the antioxidant enzymes such as SOD or CAT (35, 36). The research also revealed that excessive ROS formation and elevated levels of MDA and Pr.C, which are end-products of lipid and protein oxidation respectively, were present in the PFOA group. Additionally, the study found that the GSH content and SOD activity, which are crucial components of the body’s defense mechanism against oxidative damage, were significantly reduced in response to PFOA exposure, as shown in Table 2 (37, 38). PFOA-mediated increased ROS production is associated with impaired oxidative phosphorylation. On the other hand, PFOA-induced oxidation of the thiol groups in mitochondrial permeability transition (MPT) pores interfere with mitochondrial membrane potential and result in the release of matrix calcium, and osmotic mitochondrial swelling. These events ultimately lead to cytochrome c release from mitochondria, which initiates the internal apoptotic pathway (6, 39). Our study revealed that the PFOA treatment group showed lower mitochondrial viability, higher mitochondrial membrane potential collapse, and osmotic swelling when compared to the control group (Figure 2). Furthermore, the PFOA treatment group demonstrated a significant increase in Bax expression and a decrease in Bcl-2 expression (Figure 3).

NF-κB, a transcription factor, is a key player in regulating the expression of numerous genes that participate in inflammatory responses. This pathway is triggered by diverse stimuli, such as pathogens, environmental changes, and oxidative processes (40). Furthermore, oxidants are capable of inducing the phosphorylation of JNK, which belongs to the mitogen-activated protein kinases family and is also known as stress-activated protein kinase (41-43). The phosphorylation of c-Jun by JNK can trigger an inflammatory response, leading to the formation of active AP-1 transcription factors that transcribe genes responsible for inflammation and apoptosis, such as TNF-α (42). PFOA has been found to activate NF-κB and downstream pro-inflammatory cytokines, resulting in mast cell-mediated allergic inflammation. (44). The study found that rats administered PFOA exhibited increased expression levels of NF-κB, TNF-α, IL-6, and JNK (Figure 3). However, the administration of rutin and vitamin C supplements effectively inhibited the pro-inflammatory effects of PFOA (Figure 3). Rutin was also observed to alleviate liver damage induced by CCl_4_ by inhibiting the NF-κB, TNF-α, and cyclooxygenase-2 (COX-2) pathways (45). Additionally, rutin was found to have anti-inflammatory properties against COX-2 expression induced by ultraviolet B through modulation of MAPK signaling (16). 

Rutin as well as vitamin C has the ability to improve the harmful effects of PFOA on the liver tissue, including oxidative stress, apoptosis, and inflammation. However, the reversal was not entirely complete in some factors at the studied doses. In fact, rutin shows the capability to complete/almost complete reversal of PFOA effects on ROS formation, GSH content, JNK, Bax: Bcl2 ratio, NF-κB, and the expression of TNF-α. This was evident by these variables returning to their normal levels. In contrast, LPO, SOD activity, protein carbonylation, NO content, mitochondrial dysfunction, mitochondrial pore collapse, mitochondrial swelling, and IL-6 gene expression remained elevated in rats that were exposed to both rutin and PFOA compared to the normal group. Albeit, they were significantly lower than the corresponding levels in rats treated only with PFOA. This suggests that rutin partially restored the damaging effects of PFOA on liver function, which may account for the persistence of abnormality in the liver enzyme function (Table 1) or morphology (Figure 1). In this regard, rutin could protect against CCL_4_-induced hepatotoxicity via complete inhibition of CCL_4_-mediated overexpression of NF-κB and TNF-α (45, 46). 

This study suggests that at a dose of 100 mg/kg/day rutin was effective or more effective than vitamin C at reversing the disturbances caused by PFOA. Notably, vitamin C did not have any significant preventive effects on protein carbonylation, mitochondrial membrane potential, and JNK expression. On the other hand, rutin was able to partially alleviate the negative impact of PFOA on these variables. 

Further research is required to fully understand the extent of the protection provided, however, it appears that rutin may be more effective than vitamin C in mitigating PFOA-induced hepatotoxicity. This suggests that rutin has superior protective properties compared to vitamin C.

## Conclusion

Based on our present study, rutin could ameliorate PFOA- induced liver injury via partial restoration of oxidative damage and mitochondrial dysfunction as well as suppression of pro-inflammatory or pro-apoptotic gene expression. These findings may be useful for future application of rutin in the prevention and treatment of hepatotoxicity caused by environmental toxins.

## Authors’ Contributions

M N, M S, F T, and F S H designed the experiments; M N performed experiments and collected data; M N, M S, F S H, and S H A discussed the results and strategy; F S H supervised, directed, and managed the study; M N, M S, F T, S H A, and F S H approved the final version to be published.

## Conflicts of Interest

The authors declare that they have no conflicts of interest.
